# Exponential Slope from Absolute Lymphocyte Counts during Radio-Chemotherapy Can Predict an Aggressive Course of Cervical Cancer

**DOI:** 10.3390/cancers14205109

**Published:** 2022-10-18

**Authors:** Oyeon Cho, Mison Chun, Suk-Joon Chang

**Affiliations:** 1 Gynecologic Cancer Center, Department of Radiation Oncology, Ajou University of School of Medicine, Suwon 16499, Korea; 2 Gynecologic Cancer Center, Department of Obstetrics and Gynecology, Ajou University of School of Medicine, Suwon 16499, Korea

**Keywords:** aggressive course, cervical cancer, absolute lymphocyte count, exponential slope, radio–chemotherapy

## Abstract

**Simple Summary:**

Lymphopenia associated with clinical outcomes has three potential implications for cancer progression, treatment effects, and constitutional immune capacity. Neutrophil-to-lymphocyte ratio (NLR) and lymphopenia before and after treatment are biomarkers of immune suppression due to cancer progression and treatment effects. A decrease and an increase in the exponential slope (α) estimated from total absolute lymphocyte counts during concurrent radio–chemotherapy for cervical cancer are associated with aggressive and non-aggressive disease courses and are completely independent of stage and NLR. The association between survival and α-related mRNAs from plasma exosomes may imply an insufficient restoration of unstimulated lymphocytes in the context of immune response. The proposed α may be a practical and simple biomarker of constitutional immune capacity.

**Abstract:**

This study aimed to investigate whether the exponential slope α from absolute lymphocyte counts during concurrent radio–chemotherapy (CRT) is associated with aggressive and non-aggressive courses of cervical cancer. We analyzed 362 patients with stage IB–IVB cervical cancer treated with CRT in two groups: 323 patients without mRNA data (cohort 1) and 39 with mRNA data (cohort 2) from plasma exosomes. We calculated the α of each patient; 69 patients who died of cancer in cohort 1 were divided into 44 who died within 30 months (aggressive group), and 25 who died after more than 30 months (non-aggressive group). The median follow-up periods of cohorts 1 and 2 were 63 and 28 months, respectively. The log2 fold change (log2FC) between read counts of mRNAs before treatment and after the second week of CRT was calculated. Multivariate analyses from cohort 1 showed that neutrophil-to-lymphocyte ratio (NLR) ≥ 2.43 and α < 0.08 were statistically significant predictors of disease-specific survival (DSS) in the aggressive group (DSS-A), whereas α ≥ 0.08 was the only significant predictor of DSS in the non-aggressive group (DSS-NA). The 2.5-year DSS-A and 8-year DSS-NA rates of patients with α ≥ 0.08 and α < 0.08 were 86.7% and 73%, and 78.5% and 94.8% in the high-NLR group, respectively. In cohort 2, patients with both NLR < 2.7 and α ≥ 0.07 had a higher 2.5-year DSS rate than did those with either NLR ≥ 2.72 or α < 0.07. E2F8 and STX6 significantly correlated with ɑ and survival. The 2.5-year DSS rates in patients with E2F8 + STX6 (log2FC) < 0.2429 and ≥0.2429 were 100% and 77.2%, respectively. The exponential slope α can potentially distinguish between aggressive and non-aggressive courses in cervical cancer patients.

## 1. Introduction

Increased neutrophil-to-lymphocyte ratio (NLR), pretreatment lymphopenia, and treatment-related lymphopenia have been suggested as predictive biomarkers for survival in solid tumors [1,2,3]. Before treatment, lymphopenia can result from tumor progression or constitutional patient characteristics [4]. The apoptosis of T lymphocytes induced by tumor cells [5], decreased tumor-infiltrative lymphocytes due to programmed death-ligand 1 expressed on surfaces of antigen-presenting or tumor cells, and the suppression of immune cells such as T-helper CD4^+^ T or CD8^+^ T cells via recruiting regulatory CD4^+^ T cells can result from tumors [6,7]. However, a reduction in naïve or memory T cells may suggest short proliferation due to constitutional causes, such as a reduced thymic function or loss of the homeostasis of naïve and memory T cells sustained by T-cell receptor (TCR) signaling from contact with the major histocompatibility complex, as well as interleukins (IL) 7 and 15 [8]. Naïve B cells may also be restored through homeostatic proliferation following a lack of B cells [9]. However, NLR and absolute lymphocyte count (ALC) reflect the extent of cancer, such as the tumor stage, which is not relevant to the host’s adaptive immune response. Therefore, a simple biomarker to predict treatment results independent of cancer extent, and to reflect individual adaptive immune potential, is essential in the strategy to increase the efficacy of the current treatment through a combination of immunotherapy, such as immune checkpoint inhibitors (ICIs) or ILs overcoming an interruption of adaptive immune response by cancer or host [4].

Among T cells, which are the most radiosensitive blood cells, non-stimulated T cells are more radiosensitive than stimulated T cells, owing to the expression level of *ATM* [10,11]. In the reference range, naïve or memory T cells (36% of total ALCs) comprise more than half of all T lymphocytes (64% of total ALCs) [12]. If B cells account for 20% of the total lymphocytes and half of them are naïve or memory B cells, the proportion of naïve or memory lymphocytes among the total lymphocytes can be approximately 46%. Proliferative B cells can be more radioresistant than non-proliferative B cells are, such as T cells [13]. The increase in non-proliferative lymphocytes among total ALCs suggests that the reduction slope of ALCs may increase when the same radiation dose is delivered to peripheral lymphocytes. Therefore, estimating the slope from the lymphocyte kinetics of cancer patients treated with radiation therapy (RT) may help predict the relative deficiency of naïve and memory lymphocytes.

A pilot study of cervical cancer patients treated with weekly cisplatin-based concurrent chemoradiotherapy (CRT) reported that the log_2_ fold change (log_2_FC) of exosomal miRNAs, relevant to patients who underwent early progression within 6 months after the end of CRT, and related mRNAs revealed unresolved inflammation [14]. We hypothesized that patients who died of cervical cancer could be divided into two groups of patients with aggressive and non-aggressive courses, according to the imbalance and homeostasis of the resolution state of inflammation, which might result from poor or favorable immune capacity after standard CRT. In this context, this study aimed to investigate whether the exponential slope estimated from ALCs during CRT is associated with an aggressive or non-aggressive course of cervical cancer.

## 2. Materials and Methods

### 2.1. Patient Selection and Samples

The 442 cervical cancer patients treated with primary CRT after diagnosis with the 2018 International Federation of Gynecology and Obstetrics (FIGO) guidelines relating to stage IB–IVB cancer (converted to 2018 FIGO stage in the patients before 2018) from April 2001 to July 2020 at the Department of Radiation Oncology of Ajou University Hospital were divided into (1) a group of 400 patients without plasma exosomal RNA sequencing data before treatment and after the second week of CRT, and (2) a group of 42 patients with plasma exosomal RNA sequencing data. Then, 323 patients were retrospectively selected for the analysis of cohort 1 after 77 patients were lost to follow-up due to incomplete treatment, other treatment, or the loss of ALCs during CRT, whereas 39 were included in cohort 2 after the exclusion of 3 patients with incomplete treatment (Figure S1). Then, 24 blood samples (5–10 mL per sample) from 12 patients were collected between December 2019 and August 2020 at the Biobank of Ajou University Hospital, a member of the Korea Biobank Network, after obtaining informed consent from the patients. Next-generation sequencing (NGS) data for 60 samples from 30 patients were acquired from a previous study [15].

### 2.2. Patients

The pathological findings of all patients were confirmed by cervical biopsy. Local staging, regional lymph node (LN), and distant metastasis were evaluated using magnetic resonance imaging (MRI), computed tomography (CT), or positron emission tomography–CT. Suspicious bladder or rectal invasion was confirmed by cystoscopy or sigmoidoscopy. External beam radiotherapy (EBRT) was delivered using 10–15 MV photons to the pelvis with or without para-aortic LN. The pelvic RT dose was 45 Gy delivered in 25 fractions, and the LN lesions were boosted up to 55–60 Gy. The RT dose of metastatic sites in six patients with stage IVB was 30–55 Gy, delivered in 10–25 fractions. One patient with a single lesion in the left lung was treated with stereotactic body RT using 48 Gy in four fractions. Notably, 352 patients, except 10 replaced by EBRT, underwent high-dose-rate intracavitary brachytherapy of 4–30 Gy delivered in 1–7 fractions at point A (Iridium-192; Microselectron, Nucletron, Veenendaal, Netherlands or GammaMedplus iX, Varian, Palo Alto, CA, USA). Its location, defined by the International Commission on Radiation Units and Measurements (Report 38), was modified to 1.0–1.8 cm in patients with a small uterus. Weekly cisplatin (30–70 mg/m^2^) was administered in four to six cycles during RT in all patients. Patients were followed-up every 1–3 months after treatment completion. Primary cervical tumors, regional metastases, and distant metastases were evaluated by pelvic examination, Pap smear test, tumor markers, MRI, and CT. Among the patients with tumors persisting more than 2–6 months after CRT, four were selected to undergo salvage hysterectomy due to poor CRT response.

### 2.3. Variables

Age at diagnosis, pathology, 2018 FIGO stage, total dose (TD), NLR, pretreatment ALC (pre-ALC), all ALCs measured during CRT, overall treatment time (OT), and RT field were collected from all patients. TD was the sum of EBRT and brachytherapy dose to the central lesion (equivalent dose in 2 Gy fractions, using an alpha/beta ratio of 10 Gy). Min-ALC, the minimum ALC during CRT, was confined to a minimum value among ALCs within 40 days from the start of RT, considering the frequency of measured ALCs and pelvic irradiation over the 5 weeks (Figure S2). There were two missing values for NLR and pre-ALC in cohort 1, which were replaced by the median value of 321 patients.

### 2.4. Exponential Functions from ALCs

**Figure 1:**
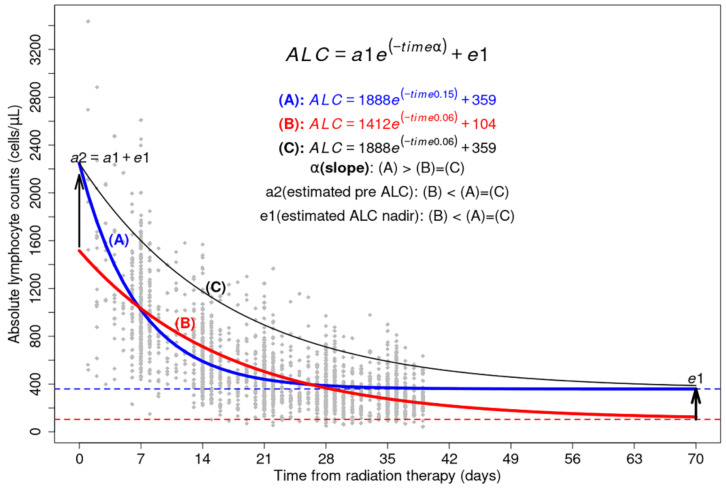
We plotted the 1927 absolute lymphocyte counts measured during radiation therapy from 323 patients (gray dots). Three estimated equations for exponential regression lines were calculated using two α, *a*1, and *e*1 values.

ALCs were available in cohorts 1 and 2 for each patient (median [range]: 5 [3–18] and 7 [4–16], respectively). These were fitted to $f\left(x\right)=a1e^{-\alpha x}+e1\;(e1>0)\;$. Among the 10 equations with the highest correlation coefficient (R^2^), the lowest α value between 0.01 and 0.2 was selected. Figure 1 presents the concept of estimated functions using the scatterplot of all ALCs of the 323 patients in cohort 1.

### 2.5. Endpoints

Disease-specific survival (DSS) was classified into two endpoints in cohort 1. We analyzed the time to death or time to progression date of 69 patients who underwent disease-specific death (DSD) using histogram, density, and scatterplots. We confirmed whether the average follow-up time for DSD could divide the 69 patients into two groups: patients with an aggressive course (DSD-A) and those with a non-aggressive course (DSD-NA). The former was called DSS for the aggressive group (DSS-A), and the latter was called DSS for the non-aggressive group (DSS-NA). The second endpoint was DSS and progression-free survival (PFS). Progression events were divided into three groups: local progression (LP), distant metastasis (DM), and both LP and DM. DSS was the only endpoint in cohort 2.

### 2.6. Log_2_ Fold Change of RNAs

NGS data from plasma exosomes, including data on small RNAs and mRNAs, were used for the analysis. After the removal of the RNAs undetected in 50% of the 84 samples, 14,908 mRNAs were analyzed. Log_2_FC values between read counts of mRNAs before treatment (control) and after the second week of CRT (treatment) were calculated after the trimmed mean of M-value normalization was calculated using edgeR for 39 patients in cohort 2. Plasma exosomal RNA sequencing and profiling were conducted by Macrogen and ROKIT Genomics (www.macrogen.com and www.rokitgenomics.com, respectively). Detailed materials and methods are presented in the Supplementary Methods.

### 2.7. Selection of mRNAs

The matrix of Pearson’s correlations among all mRNAs, α, NLR, progression, and DSD was calculated using the recorr function in the Hmisc package. The mRNAs associated with either α or DSD or both, or both NLR and DSD were selected. The optimal model using selected mRNAs was suggested by an exhaustive search of regsubsets in the leaps package. The sum and difference of mRNAs in the suggested model were found to be relevant to the DSD using the Wilcoxon rank-sum test.

### 2.8. Survival Analysis

We compared the differences between age at diagnosis, pathology, 2018 FIGO stage, OT, RT field, TD, pre-ALC, min-ALC, NLR, *a*2 (estimated pre-ALC), *e*1 (estimated ALC nadir), and whether the survival events occurred between the two groups of patients according to the median value of α or NLR using the χ^2^ test for categorical data and *t*-test or Wilcoxon rank-sum test according to the normality test for continuous data in cohort 1. Log_2_FC values of mRNAs were added to this comparison in cohort 2. We performed univariate and multivariate analyses for DSS, PFS, DSS-A, DSS-NA, and DSS-A in patients with stage IIIC2–IVB disease and DSS-A in those with stage IIIA–IIIC1 disease using the Cox proportional hazards model (cohort 1). We performed univariate analyses as a reference (Figure S3) and multivariate analyses using backward elimination, including all candidate variables except a noise variable. The 2.5-year DSS-A (2.5DSS-A), 8-year DSS-NA (8DSS-NA), and 8-year DSS (8DSS) between the two groups of patients according to the median value of α, NLR, and both α and NLR were compared using Kaplan–Meier curves and log-rank tests (cohort 1). The 2.5-year DSS (2.5DSS) between the two groups of patients according to the median value of selected mRNAs was compared using the Kaplan–Meier method (cohort 2).

All data analysis and visualization were performed using R version 4.1.1 (https://www.r-project.org, accessed on December 2021).

## 3. Results

### 3.1. Concept of Exponential Functions from ALCs

**Figure 2:**
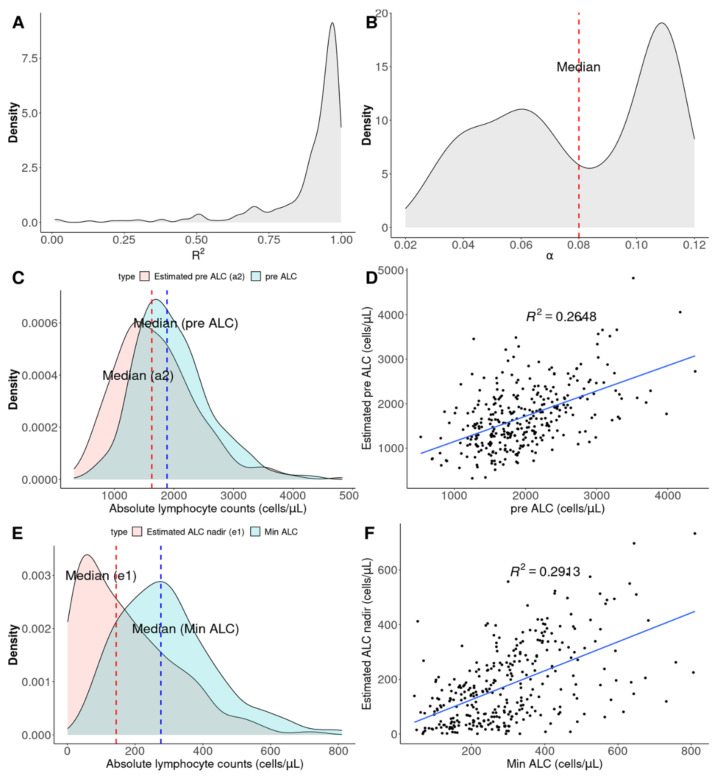
Characteristics of exponential functions ($ALC=a1e^{-\alpha time}+e1$) fitted by ALCs from each of the 323 patients. Density plots for (**A**) R^2^ and (**B**) α (slope) of fitted functions. (**C**) Density plot and (**D**) scatterplot to compare pre-ALCs with estimated pre-ALCs (*a*2 = *a*1 + *e*1). (**E**) Density plot and (**F**) scatterplot to compare min-ALCs with estimated ALC nadirs (*e*1). ALC—absolute lymphocyte count.

Figure 1 presents two estimated functions with the lowest α (A: blue line) and highest α (B: red line) and a reference function (C: black line) with the same *a*1 and *e*1 as the blue line and α as the red line. α means the slope of the exponential function. *a*2, the sum of *a*1 and *e*1, presents the pre-ALC estimated from ALCs during CRT, and *e*1 is the estimated ALC nadir when RT is continued. The four density plots for R^2^, α, *a*2, and *e*1, and two scatterplots between *a*2 and pre-ALC and between *a*1 and min-ALC for each patient are presented in Figure 2 (cohort 1). These plots support the acceptable correlation of estimated functions of the two groups according to the median value of α, the association between pre-ALC and *a*2, and the correlation between min-ALC and *e*1.

### 3.2. Two Types of Disease Courses

**Figure 3:**
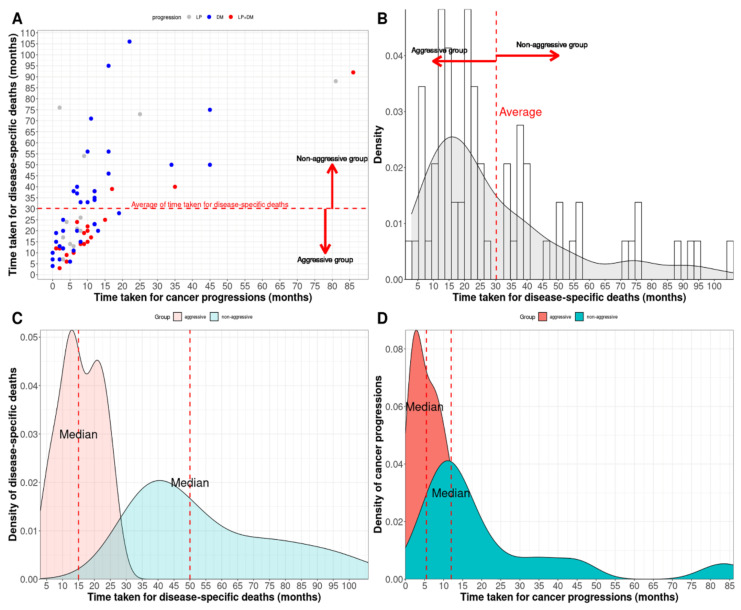
The 69 patients who underwent disease-specific death (DSD) were divided into the aggressive (44 patients) and non-aggressive (25 patients) groups according to the average time taken for DSD. (**A**) Scatterplot between the time to progression and the time to DSD after the end of treatment (local progression (LP)—gray; distant metastasis (DM)—blue; LP + DM—red) and (**B**) density plot and histogram of the time 69 patients underwent DSD. Density plots between the aggressive and non-aggressive groups for (**C**) DSD and (**D**) cancer progression, respectively.

In total, 44 of the 69 patients, defined as DSD-A, died of cervical cancer before 30 months, whereas the other 25 patients, defined as DSD-NA, died after 30 months, the average follow-up time for DSDs. These data show that DSD-A includes patients with rapid DSD (median: 15 months) after early progression (median: 6 months), containing a relatively high proportion of both LP and DM, whereas DSD-NA includes those with delayed DSD (median: 50 months), despite early progression (median: 12 months), with a relatively high proportion of DM (Figure 3 and Table S1).

### 3.3. Survival Analysis (Cohort 1)

**Figure 4:**
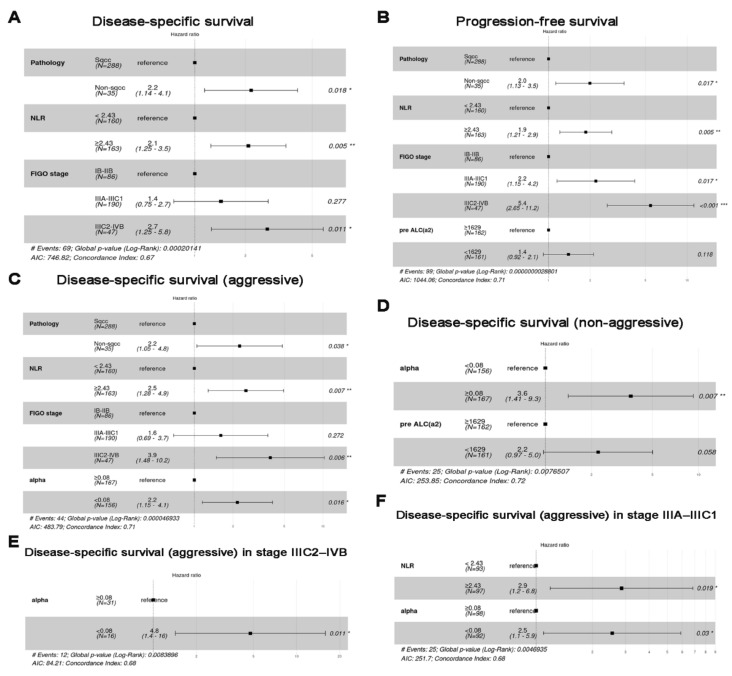
Multivariate analysis using the Cox regression model of (**A**) disease-specific survival (DSS), (**B**) progression-free survival, (**C**) DSS (aggressive group), (**D**) DSS (non-aggressive group), (**E**) DSS (aggressive group) in stage IIIC2–IVB, and (**F**) DSS (aggressive group) in stage IIIA–IIIC1. NLR—neutrophil-to-lymphocyte ratio; Sqcc—squamous cell carcinoma; FIGO—International Federation of Gynecology and Obstetrics; AIC—Akaike information criterion.; * *p* < 0.05, ** *p* < 0.01, *** *p* < 0.001.

Table 1 describes the 323 patients’ characteristics and compares patients with α ≥0.08 and with <0.08. Patients with low α included significantly more patients with adenocarcinoma (AC) or adenosquamous cell carcinoma (ASC), DSD-A, low *a*2, and low *e*1, whereas they included fewer patients with DSD-NA than they did patients with high α. Multivariate analyses showed that α<0.08 and α≥0.08 were independent and significant predictors of DSS-A and DSS-NA, respectively (Figure 4C–F).

**Figure 5:**
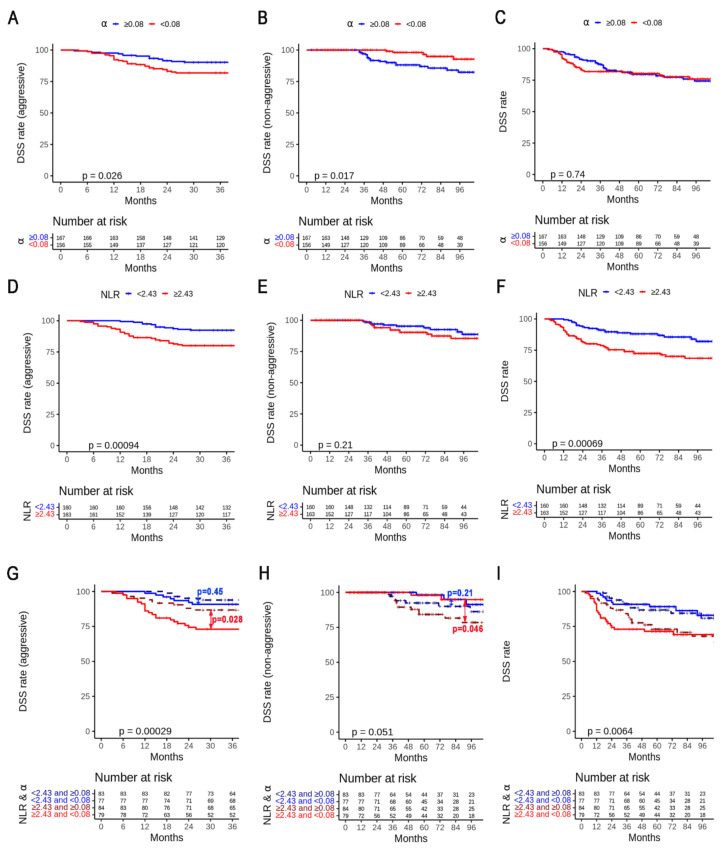
(**A**,**D**,**G**) Kaplan–Meier plots and log-rank tests of disease-specific survival (DSS) in the aggressive group, (**B**,**E**,**H**) DSS in the non-aggressive group, and (**C**,**F**,**I**) DSS according to median values of α, NLR, and both α and NLR, respectively. NLR—neutrophil-to-lymphocyte ratio.

However, α was not a significant predictor of DSS. NLR ≥ 2.43, non-squamous cell carcinoma, and FIGO stage were significant predictors of DSS, PFS, and DSS-A, but were not associated with DSS-NA (Figure 4A–C). In subgroup analysis, a high NLR was a significant predictor of DSS-A in stage IIIA–IIIC1 (Figure 4F). There were no significant predictors of stage IB–IIB. The median follow-up duration was 63 months. In cohort 1, the 2.5DSS-A, 8DSS-NA, 8DSS, and 8PFS rates were 86.2%, 87.2%, 75.2%, and 65.7%, respectively, for all patients. The patients with α ≥ 0.08 had a 2.5DSS-A rate of 90.3%, whereas those with α < 0.08 had a 2.5DSS-A rate of 81.8% (*p* = 0.026) (Figure 5A).

Conversely, the patients with α ≥ 0.08 had an 8DSS-NA rate of 82.4%, whereas those with α < 0.08 had an 8DSS-NA rate of 92.8% (*p* = 0.017) (Figure 5B). However, there was no significant difference in the 8DSS rate between the two groups (*p* = 0.74) (Figure 5C). Patients with NLR ≥ 2.43 had significantly lower 2.5DSS-A and 8DSS rates than those of patients with NLR < 2.43 (80.1% and 92.4%, *p* < 0.001 and 68.5% and 82.0%, *p* < 0.001, respectively) (Figure 5D,F). However, there was no significant difference in 8DSS-NA rate between the high- and low-NLR groups (85.5% and 88.7%, respectively, *p* = 0.21) (Figure 5E). The 2.5DSS-A rates of α ≥ 0.08 and α < 0.08 were 86.7% (*n* = 84) and 73.0% (*n* = 79) and 93.9% (*n* = 83) and 90.8% (*n* = 77) in the high- (*p* = 0.028) and low-NLR (*p* = 0.45) groups, respectively (Figure 5G). The 8DSS-NA rates of α ≥ 0.08 and α < 0.08 were 78.5% and 94.8% and 86.2% and 91.3% in the high- (*p* = 0.046) and low-NLR (*p* = 0.21) groups, respectively (Figure 5H). However, there was no significant difference in the 8DSS rate between the two groups in high- and low-NLR groups (Figure 5I).

### 3.4. Selected mRNAs and Survival Analysis (Cohort 2)

**Figure 6:**
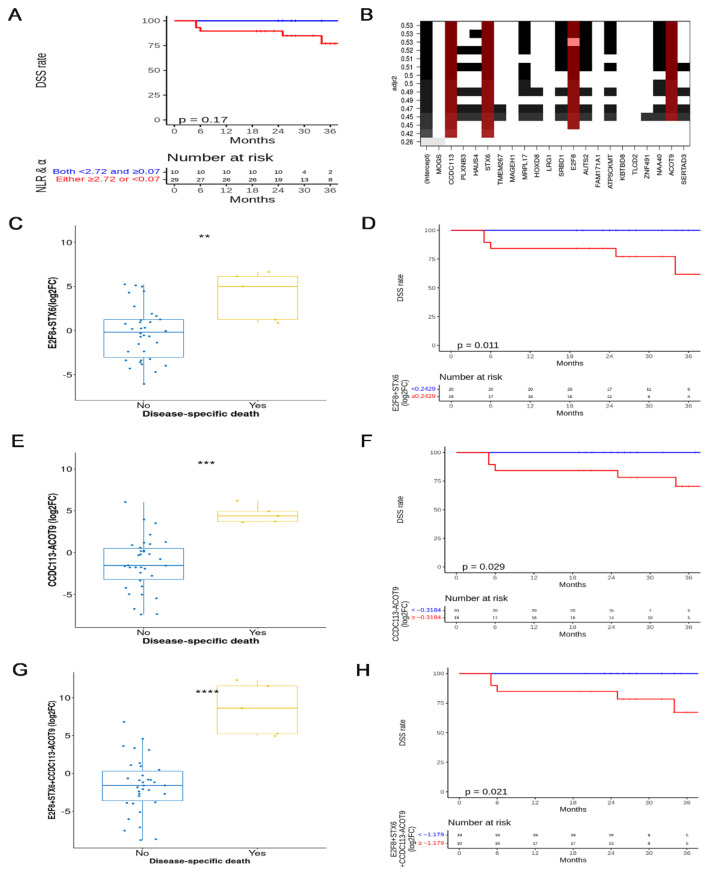
(**A**) Kaplan–Meier plot and log-rank test of disease-specific survival (DSS) according to the median value of both α and NLR in cohort 2. (**B**) CCDC113, STX6, E2F8, and ACOT9 were included in most of the cases to predict DSS with the subsets of 21 mRNAs from plasma exosomes, relevant to both survival and either α or NLR (red rectangles). (**C**) The sum of E2F8 and STX6 relevant to both survival and α; (**E**) CCDC113-ACOT9 relevant to both survival and NLR; and (**G**) E2F8 + STX6 + CCDC113-ACOT9 relevant to survival, α, and NLR showed a significant difference between the two groups according to whether the patients died of cervical cancer or not, respectively. Kaplan–Meier plots and log-rank tests of DSS (**D**) according to the median value of E2F8 + STX6 (**F**) according to the median value of CCDC113-ACOT9 and (**H**) according to the median value of E2F8 + STX6 + CCDC113-ACOT9. NLR—neutrophil-to-lymphocyte ratio; ** *p* < 0.01, *** *p* < 0.001, **** *p* < 0.0001.

Table S2 describes the 39 patients’ characteristics and compares patients with both NLR < 2.72 and α≥0.07 with those with either NLR ≥2.72 or α<0.07. The median follow-up duration was 28 months. The 2.5DSS rate was 89.0% in all patients. Patients with both low NLR and high α had a higher 2.5DSS rate than those of patients with either high NLR or low α (100% vs. 84.9%, *p* = 0.17) (Figure 6A).

The ratio of adjusted R^2^ to the number of selected mRNAs was the highest when 4 of the 21 mRNAs, correlated with α or NLR to predict DSS, were selected (Figure S4). The four mRNAs of E2F8, STX6, CCDC113, and ACOT9 were core members of various subsets consisting of 2–15 mRNAs in Figure 6B. E2F8 and STX6 were relevant to α, whereas CDC113 and ACOT9 were associated with NLR. The E2F8 (log_2_FC) of 11 patients was zero, whereas the STX6 (log_2_FC) of all patients was non-zero. The CCDC113 (log_2_FC) of seven patients was zero, whereas the ACOT9 (log_2_FC) of all patients was non-zero. The patients who underwent DSD had higher E2F8 *+* STX6 (log_2_FC) than those of the patients who did not, and the 2.5DSS rates of patients with E2F8 + STX6 (log_2_FC) < 0.2429 and those with ≥0.2429 were 100% and 77.2%, respectively (*p* = 0.011) (Figure 6C,D). Patients who underwent DSD had a higher CCDC113-ACOT9 (log_2_FC) than patients who did not, and there were significant differences in 2.5DSS rate between the two groups according to the median value of CCDC113-ACOT9 (log_2_FC) (<−0.3184 vs. ≥−0.3184: 100% vs. 78.2%, *p* = 0.029) (Figure 6E,F). Patients who underwent DSD had higher E2F8 + STX6 + CCDC113-ACOT9 (log_2_FC) than patients who did not, and the 2.5DSS rates of E2F8 + STX6 + CCDC113-ACOT9 (log_2_FC) < −1.179 and ≥−1.179 were 100% and 78.46%, respectively (*p* = 0.021) (Figure 6G,H). Patients who underwent DSD had higher STX6-ACOT9 (log_2_FC) than those of patients who did not, and there were significant differences in 2.5DSS rate between the two groups according to the median value of STX6-ACOT9 (log_2_FC) (<0.3657 vs. ≥0.3657: 100% vs. 77.7%, *p* = 0.021) (Figure S5).

## 4. Discussion

The present study revealed that the α slope of exponential function estimated from the serial ALCs of each patient could differentiate between patients with aggressive and non-aggressive courses in cervical cancer treated with CRT by confirming that low and high α could predict DSS-A and DSS-NA, respectively. Furthermore, α can be utilized to identify potential biological biomarkers to predict cervical cancer patients with aggressive course, as we confirmed that the sum of both E2F8 and STX6, two mRNAs from plasma exosomes correlated with α, was associated with DSS in cohort 2.

This study demonstrated a significant difference in that DSS-A and DSS-NA, instead of DSS, as primary endpoints were used for survival analysis. The utilization of two endpoints was based on the assumption that high or low immune capacities of patients, as a host factor of lymphopenia, can influence treatment results. The association between DSS-A (or DSS-NA) and α supports this assumption. α might be an independent prognostic factor associated with the durability of the immune system, aside from cancer extent, such as stage or NLR (Table 1 and Table S3). Patients with high α underwent delayed DSD, despite some of these patients experiencing early metastasis, whereas those with low α rapidly died of cervical cancer. α potentially meant that the ratio of stimulated and unstimulated lymphocytes was constant in normal immune capacity. During the fierce attack of cancer on multiple large normal tissues, the pool of unstimulated lymphocytes may be consumed through the rapid transition from naïve to effector lymphocytes, but can be restored repeatedly [8,16]. The reduction in lymphocytes via RT in such an environment increases the immune capacity to restore unstimulated lymphocytes to the test. If an individual has a lower immune capacity, the ratio of unstimulated to stimulated lymphocytes in the blood decreases. Fortunately, stimulated T cells are three times more radioresistant than unstimulated T cells within 48 h of 1 Gy [11]. Moreover, plasma cells are radioresistant in a dose-dependent manner, whereas naïve B cells show dose-dependent radiosensitivity [13]. This difference may have influenced the slopes between patients with low and high immune capacities. Immune suppression by cancer is related to a decrease in TCR diversity and a decrease in activated CD4+ CD8+ lymphocytes, whereas a patient’s immune capacity refers to a physical ability to sufficiently replenish these lymphocytes when they are exhausted [4]. Several recent studies have reported that high levels of peripheral naïve and memory T cells are relevant to good prognosis and immunotherapy effectiveness in multiple cancers, including non-small cell lung cancer, melanoma, and pediatric cancer, supporting the importance of restoring unstimulated lymphocytes [17,18,19].

In addition, IL treatments based on this concept restore CD8+ memory T cells in IL-15 and naïve and memory CD4+/CD8+ subsets in IL-7, but do not show significant improvement in treatment outcomes [4]. Recently, clinical trials have been undertaken to improve treatment outcomes using IL-7 or IL-15 and ICIs in combination in various cancers (NCT03901573, NCT04594811, NCT04332653, NCT04984811) [20]. In this context, α, which is a practical marker that can discriminate whether immune capacity is reduced, has clinical significance in specifying an immunotherapy group of cervical cancer patients. If the disease has progressed sufficiently to suppress a patient’s immune function, a low immune capacity can quickly lead to life-threatening results, even with appropriate treatment. Patients with high NLR were strongly associated with lymphopenia resulting from tumor progression (Table S3). Among them, patients with low α died quickly, whereas those with high α died of cervical cancer. This result suggests that α is an independent prognostic factor relevant to host immune capacity. AC or ASC pathology was not relevant to a high NLR, but to a low α. Therefore, the reason why patients with AC have poorer clinical outcomes than those of squamous cell carcinoma patients treated with CRT may be due to constitutional immune capacity [21].

Furthermore, mRNAs from extracellular vesicles, including exosomes, which might be important for the modulation of innate and adaptive immune responses [22], were investigated. Cohort 2 suggests that E2F8 and STX6 are exosomal mRNAs that are relevant to α. E2F8, which is abundant in LNs and plasma cells, is a cell cycle regulator associated with B-cell activation [23,24]. This can be interpreted as a relative increase owing to a decrease in the number of non-activated B cells. STX6, which constitutes the SNARE complex, increases tumor necrosis factor (TNF)-α exocytosis in activated macrophages [25,26]. TNF-α induces the apoptosis of activated cytotoxic T cells and increases naïve and memory T cells while accumulating regulatory T cells [27]. NLR-associated exosomal mRNAs CCDC113 and ACOT9 were also associated with treatment outcomes. CCDC113, which is abundant in basophils, is hypothesized to be associated with pro-inflammation; however, no related studies have been reported regarding this. ACOT9, which is abundant in monocytes, is an acyl-coenzyme A (CoA) thioesterase group that degrades acyl-CoA into CoA and fatty acids. Fat accumulation due to ACOT9 deficiency in tumor-associated macrophages may promote tumor growth and suppress immunity [28]. The four mRNAs proposed in this study may reflect the implications of α in a host immune capacity and NLR as an immune-suppression effect of cancer, from the perspective of past studies related to immune cells.

In the present study, α has four clinical effects. First, to our knowledge, this is the first study to demonstrate that α is an intrinsic prognostic factor in cervical cancer patients treated with CRT, independent of disease progression. Second, in the context of the high interest in the clinical study results of the combined treatment with IL-7 or IL-15 and ICIs in advanced cancers, α can directly suggest a subgroup that can expect a good treatment response to immunotherapy. Third, it is practical and economical in that the treatment result can be predicted while proceeding with the usual treatment without additional examination. Fourth, α, as a clinical marker for differentiating host factors from cancer factors, may guide the identification of various meaningful genetic biomarkers.

However, this study has the following limitations. First, as this was a retrospective study, a large amount of data were excluded due to a lack of follow-up for each patient. Second, there was no subset analysis to distinguish between stimulated and unstimulated lymphocytes, such as effector and naïve T cells. Third, there was insufficient evidence regarding the expression sites of the suggested mRNAs and their roles. Fourth, although cohort 2 was presented to conform to α, a study with a larger group is required for validation because the group size was small and the follow-up period was short. To compensate for these limitations, a large-scale prospective study involving multicenter and small-scale translational studies is required. In a small-scale translational study, it is necessary to identify unstimulated and stimulated lymphocytes from a patient’s blood and to prove their correlation with α through RNA sequencing data for immune cell-derived exosomes. Separately, data from cervical cancer patients who underwent CRT at multiple centers should be collected to determine whether α can predict patient populations with an aggressive disease course. In addition, it is necessary to review the proposed assumptions by examining the treatment response of subgroups according to α in clinical studies related to the treatment of ICI or IL.

## 5. Conclusions

In the present study, we suggest that α can specify an individual immune capacity among three factors of lymphopenia, including cancer and treatment, based on data that could distinguish between aggressive and non-aggressive courses in cervical cancer patients treated with CRT. This may be utilized as a simple and practical biomarker to examine individual immune capacity if evidence is generated through additional studies in the near future.

## Figures and Tables

**Table 1:** Patients’ characteristics according to the median value of alpha (cohort 1).

Factors	Median [IQR]	ALL (*n* = 323)		*p*
α ($ALC=a1e^{-\alpha time}+e1$)	0.08 [0.055–0.11]	≥0.08 (*n* = 167)	<0.08 (*n* = 156)	
Age (years at diagnosis)	57 [48–69]			0.36
≥50	223 (69.0%)	111 (66.5%)	112 (71.8%)	
<50	100 (31.0%)	56 (33.5%)	44 (28.2%)	
Pathology				0.011
Adenocarcinoma	20 (6.2%)	8 (4.8%)	12 (7.7%)	
ASC	12 (3.7%)	2 (1.2%)	10 (6.4%)	
Carcinoma	3 (0.9%)	0 (0%)	3 (1.9%)	
Squamous cell carcinoma	288 (89.2%)	157 (94.0%)	131 (84.0%)	
FIGO stage				0.056
IB–IIB	86 (26.6%)	38 (22.8%)	48 (30.8%)	
IIIA–IIIC1	190 (58.8%)	98 (58.7%)	92 (59.0%)	
IIC2–IVB	47 (14.6%)	31 (18.6%)	16 (10.3%)	
Treatment time (days)	53 [49–55]	53.0 [49.0–58.0]	53.0 [49.0–60.0]	0.946
Radiation therapy field				0.026
Pelvis and PALN	63 (19.5%)	41 (24.6%)	22 (14.1%)	
Pelvis	260 (80.5%)	126 (75.5%)	134 (85.9%)	
Total dose (EQD2)	70.2 [68.1–73.2]	69.8 [68.0–73.4]	70.4 [68.1–72.9]	0.976
Pre-ALC (cells/μL)	1884 [1543–2326]			0.166
≥1884	160 (49.5%)	76 (45.5%)	84 (53.9%)	
<1884	163 (50.5%)	91 (54.5%)	72 (46.2%)	
Min-ALC (cells/μL)	276 [193–377]			0.288
≥276	163 (50.5%)	79 (47.3%)	84 (53.9%)	
<276	160 (49.5%)	88 (52.7%)	72 (46.2%)	
Neutrophil-to-lymphocyte ratio	2.43 [1.41–3.35]			1
<2.43	160 (49.5%)	83 (49.7%)	77 (49.4%)	
≥2.43	163 (50.5%)	84 (50.3%)	79 (50.6%)	
*a*2 = *a*1 + *e*1 (cells/μL)	1629 [1237–2108]			0
≥1629	162 (50.2%)	105 (62.9%)	57 (36.5%)	
<1629	161 (49.9%)	62 (37.1%)	99 (63.5%)	
*e*1 (cells/μL)	144 [63–261]			0
≥144	163 (50.5%)	128 (76.7%)	35 (22.4%)	
<144	160 (49.5%)	39 (23.4%)	121 (77.6%)	
Progression				0.111
No	224 (69.4%)	111 (66.5%)	113 (72.4%)	
Locoregional progression (LP)	23 (7.1%)	14 (8.4%)	9 (5.8%)	
Distant metastasis (DM)	54 (16.7%)	34 (20.4%)	20 (12.8%)	
LP + DM	22 (6.8%)	8 (4.8%)	14 (9.0%)	
DSD (aggressive)				0.043
No	279 (86.4%)	151 (90.4%)	128 (82.1%)	
Yes	44 (13.6%)	16 (9.6%)	28 (18.0%)	
DSD (non-aggressive)				0.02
No	298 (92.3%)	148 (88.6%)	150 (96.2%)	
Yes	25 (7.7%)	19 (11.4%)	6 (3.9%)	
DSD				0.962
No	254 (78.6%)	132 (79.0%)	122 (78.2%)	
Yes	69 (21.4%)	35 (21.0%)	34 (21.8%)	

IQR—interquartile range; ALC—absolute lymphocyte count; ASC—adenosquamous cell carcinoma; FIGO—International Federation of Gynecology and Obstetrics; PALN—para-aortic lymph node; EQD2—equivalent dose in 2 Gy fractions; Pre-ALC—ALC before treatment; Min-ALC—minimum ALC during radio–chemotherapy; *a*2—estimated pre-ALC; *e*1—estimated ALC nadir; DSD—disease-specific death.

## Data Availability

All data are available in the following forms: raw sequencing data: ArrayExpress (accession numbers: E-MTAB-10215, 10930, 12187); coding and dataset: https://github.com/oyeoncho/alpha accessed on 24 September 2022).

## Supplementary Materials

The following supporting information can be downloaded at: https://www.mdpi.com/article/10.3390/cancers14205109/s1, Figure S1: Process of selecting the two cohorts; Figure S2: Number of absolute lymphocyte count measurements; Figure S3: Univariate regression analysis; Figure S4: Adjusted R^2^ of regression analysis between disease-specific survival and log_2_ fold change values of mRNAs selected among 21 mRNAs from plasma exosomes, relevant to both survival and either ɑ or neutrophil-to-lymphocyte ratio, were plotted, and the regression by four selected mRNAs was most efficient to predict disease-specific survival (red line); Figure S5: (A) The subtract of *STX6* and *ACOT9* showed a significant difference between the two groups according to whether the patients died of cervical cancer or not, respectively. (B) Kaplan–Meier plots and log-rank tests of disease-specific survival according to the median value of the *STX6-ACOT9*; Table S1: Comparison between the aggressive and non-aggressive groups (cohort 1); Table S2: Patients’ characteristics (cohort2); Table S3: Patients’ characteristics according to the median value of neutrophil-to-lymphocyte ratio (cohort 1); Supplementary Methods: sample preparation and NGS procedure.
