# Supplementary material for: Exponential Slope from Absolute Lymphocyte Counts during Radio-Chemotherapy Can Predict an Aggressive Course of Cervical Cancer
Source: Cancers (Basel). 2022 Oct 18;14(20):5109. doi: 10.3390/cancers14205109 (PMC9600990; doi:10.3390/cancers14205109)
Supplement: Supplementary file 1 [file cancers-14-05109-s001.zip › cancers-1961362-supplementary.pdf]

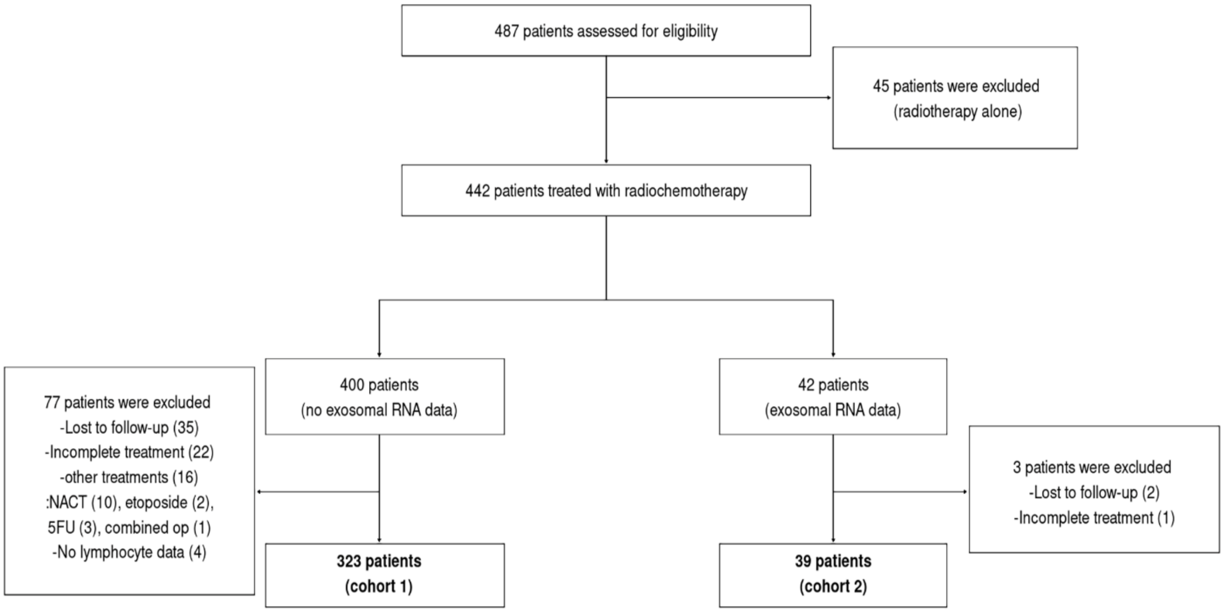

Figure S1. Process of selecting the two cohorts. NACT, neoadjuvant chemotherapy; 5FU, 5-fluorouracil

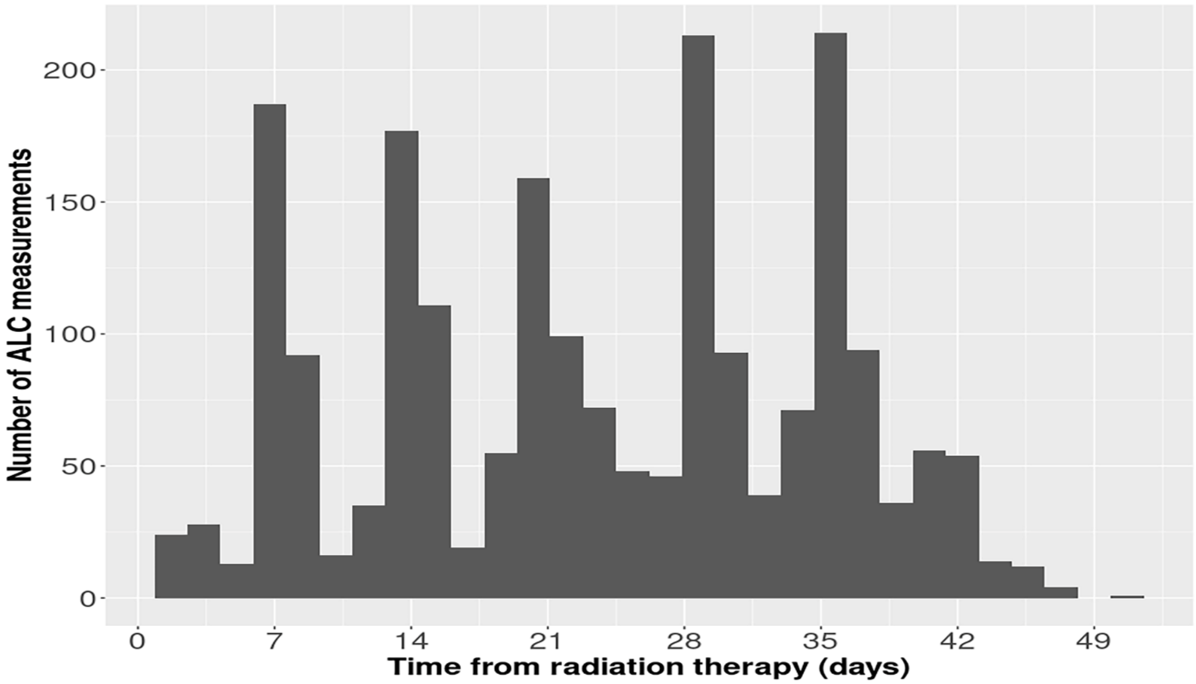

Figure S2. Number of absolute lymphocyte count measurements at the time of radiation therapy

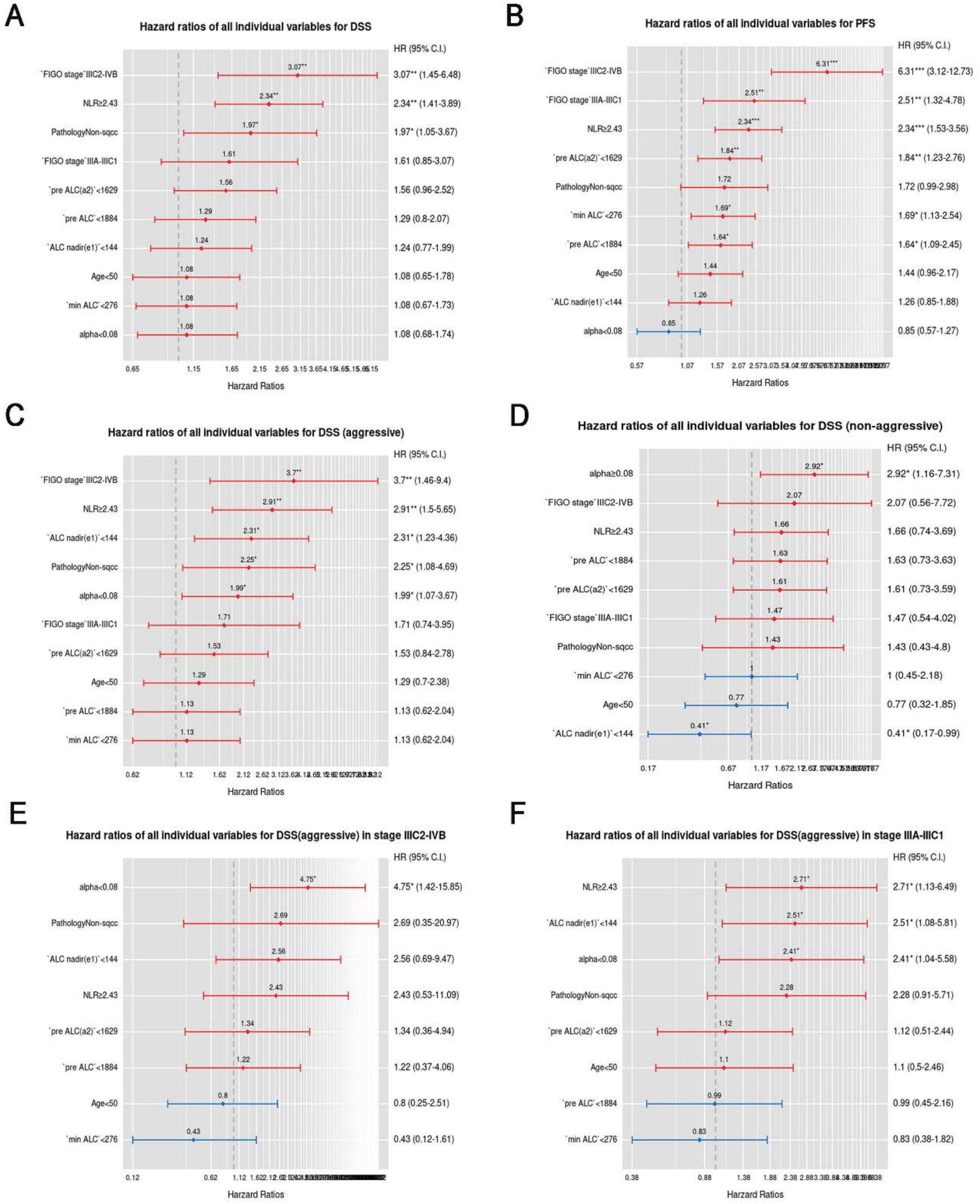

Figure S3. Univariate regression analysis for (A) disease-specific survival, (B) progression-free survival, (C) disease-specific survival (aggressive group), (D) disease-specific survival (non-aggressive group), (E) disease-specific survival (aggressive group) in stage IIIC2–IVB, and (F) disease-specific survival (aggressive group) in stage IIIA–IIIC1; * *p* <0.05, ***p* <0.01, ****p* <0.001

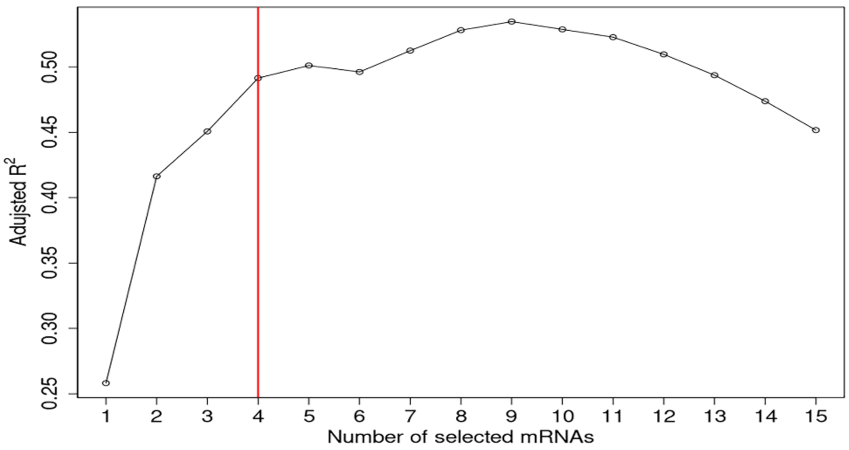

Figure S4. Adjusted R^2 of regression analysis between disease-specific survival and $\log_2$ fold change values of mRNAs selected among 21 mRNAs from plasma exosomes, relevant to both survival and either α or neutrophil-to-lymphocyte ratio, were plotted, and the regression by four selected mRNAs was most efficient to predict disease-specific survival (red line)

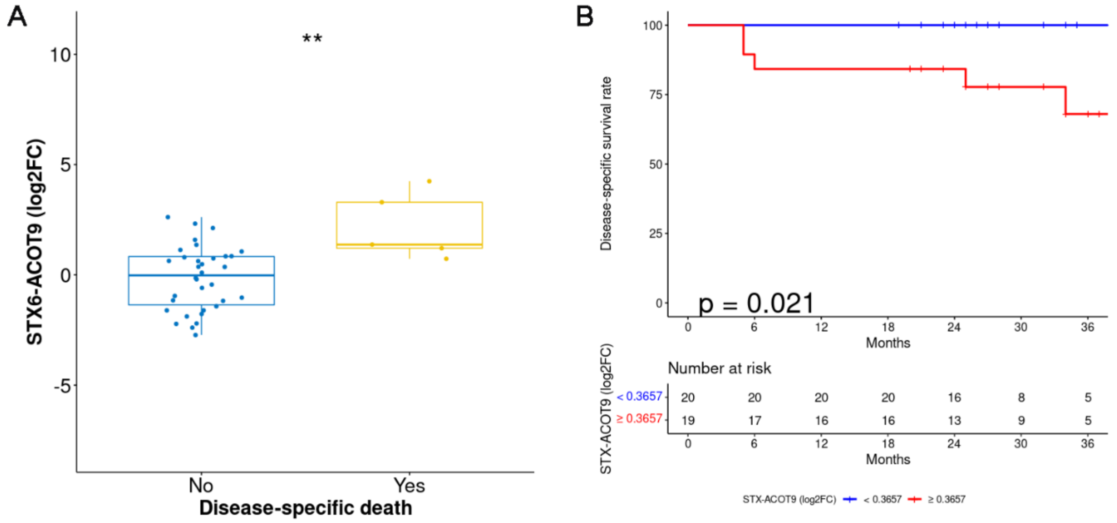

Figure S5. (A) The subtract of STX6 and ACOT9 showed a significant difference between the two groups according to whether the patients died of cervical cancer or not, respectively. (B) Kaplan–Meier plots and log-rank tests of disease-specific survival according to the median value of the STX6-ACOT9; **$p < 0.01$

Table S1. Comparison between the aggressive and non-aggressive groups in the 69 patients who underwent disease-specific death (cohort 1)

Factors	Disease-specific death (n=69)		*P*
	Non-aggressive (n=25)	Aggressive (n=44)	
Age (years at diagnosis)			0.658
≥50	18 (72.0%)	28 (63.6%)	
<50	7 (28.0%)	16 (36.4%)	
Progression			0.019
Unknown	2 (8.0%)	0 (0.0%)	
Locoregional progression (LP)	4 (16.0%)	8 (18.2%)	
Distant metastasis (DM)	17 (68.0%)	20 (45.5%)	
LP+DM	2 (8.0%)	16 (36.4%)	
FIGO stage			0.558
- IB–IIB	5 (20.00%)	7 (15.91%)	
- IIIA–IIIC1	16 (64.00%)	25 (56.82%)	
- IIIC2–IVB	4 (16.00%)	12 (27.27%)	
	Mean±standard deviation		
Neutrophil-to-lymphocyte ratio	2.80±1.59	3.43±1.88	0.165
Pre ALC (cells/μL)	1853.13±510.26	1976.02±773.49	0.431
Min ALC (cells/μL)	295.64±158.61	257.07 ±128.64	0.276
a2=a1+e1 (cells/μL)	1569.96±690.54	1512.34±675.55	0.737
e1 (cells/μL)	236.28±139.91	136.30±133.35	0.004
α	0.09±0.03	0.07±0.03	0.005

FIGO, International Federation of Gynecology and Obstetrics; ALC, absolute lymphocyte count; Pre ALC, ALC before treatment; Min ALC, minimum ALC during radiochemotherapy; a2, estimated pre ALC; e1, estimated ALC nadir

Table S2. Patients' characteristics according to the median value of alpha and neutrophil-to-lymphocyte ratio (cohort 2)

Factors	Median [IQR] or Mean±SD	ALL (n=39)		*P*
α<br>NLR	0.07 [0.05–0.11]<br>2.72 [1.84–3.7]	Both ≥0.07 and <2.72 (n=10)	Either <0.07 or ≥2.72 (n=29)	
Age (years at diagnosis)	50 [45–60]			1
≥50	23 (59.0%)	6 (60.0%)	17 (58.6%)	
<50	16 (41.0%)	4 (40.0%)	12 (41.4%)	
Pathology				0.325
Adenocarcinoma	6 (15.4%)	0 (0.0%)	6 (20.7%)	
ASC	1 (2.6%)	0 (0.0%)	1 (3.5%)	
Carcinoma	1 (2.6%)	0 (0.0%)	1 (3.5%)	
Sqcc	31 (79.5%)	10 (100.0%)	21 (72.4%)	
FIGO stage				0.71
- IB–IIB	10 (25.6%)	2 (20.0%)	8 (27.6%)	
- IIIC1	19 (48.7%)	6 (60.0%)	13 (44.8%)	
- IIIC2–IVB	10 (25.6%)	2 (20.0%)	8 (27.6%)	
Treatment time	53 [49-55]	53.0 ± 2.6	55.0 ± 4.6	0.2
Radiation therapy field				0.482
- Pelvis and PALN	9 (23.1%)	1 (10.0%)	8 (27.6%)	
- Pelvis	39 (76.9%)	9 (90.0%)	21 (72.4%)	
Total dose (EQD2)	76.3 [72.3–81.8]	76.3 [76.3–76.3]	76.3 [72.3–81.8]	0.826
Pre ALC (cells/µL)	1746 [1793–2009]			0.232
≥1746	19 (48.7%)	7 (70.00%)	12 (41.4%)	
<1746	20 (51.3%)	3 (30.00%)	17 (58.6%)	
Min ALC (cells/µL)	203 [157–308]			1
≥276	20 (51.3%)	5 (50.0%)	15 (51.7%)	
<276	19 (48.7%)	5 (50.0%)	14 (48.3%)	
a2=a1+e1 (cells/µL)	1393 [1033–1772]			0.082

≥1629	20 (51.3%)	8 (80.0%)	12 (41.4%)	
<1629	19 (48.7%)	2 (20.0%)	17 (58.6%)	
e1 (cells/μL)	101 [50–177]			0.785
≥144	20 (51.3%)	6 (60.00%)	14 (48.3%)	
<144	19 (48.7%)	4 (40.00%)	15 (51.7%)	
Progression				0.623
No	30 (76.9%)	9 (90.0%)	21 (72.4%)	
Locoregional progression (LP)	1 (2.6%)	0 (0.0%)	1 (3.5%)	
Distant metastasis (DM)	5 (12.8%)	1 (10.0%)	4 (13.8%)	
LP+DM	3 (7.7%)	0 (0.0%)	3 (10.3%)	
DSD				0.391
No	34 (87.2%)	10 (100.0%)	24 (82.8%)	
Yes	5 (12.8%)	0 (0.0%)	5 (17.2%)	
Log_2 fold change of exosomal mRNA between before treatment and second week of radiation therapy				
E2F8	0 [–2.66–1.18]/ –0.11±2.98	–1.81±2.00	0.48±3.06	0.034
STX6	0.29 [–0.13–0.86]/0.36±0.89	0.13 [–0.33–0.48]	0.32 [–0.03–0.88]	0.219
E2F8+STX6	0.24 [–2.38–1.78]/0.25±3.3	–1.80±1.77	0.96±3.42	0.003
CCDC113	0 [–2.11–1.15]/ –0.37±3.09	–0.65±1.93	–0.27±3.43	0.739
ACOT9	0.15 [–0.2–1.18]/0.23±1.27	0.63±1.06	0.09±1.32	0.243
CCDC113-ACOT9	–0.32[–2.59–1.22]/ –0.59±3.56	–1.29±1.80	–0.35±4.00	0.325
E2F8+STX6+CCDC113-ACOT9	–1.18 [–2.87–2.23]/ –0.34±4.97	–3.09±2.19	0.61±5.32	0.004

IQR, interquartile range; SD, standard deviation; ALC, absolute lymphocyte count; NLR, neutrophil-to-lymphocyte ratio; Sqcc, squamous cell carcinoma; ASC, adeno-Sqcc; FIGO, International Federation of Gynecology and Obstetrics; PALN, para-aortic lymph node; EQD2, equivalent dose in 2 Gy fractions; Pre ALC, ALC before treatment; Min ALC, minimum ALC during radiochemotherapy (<40 days); a2, estimated pre ALC; e1, estimated ALC nadir; DSD, disease-specific death

Table S3. Patients' characteristics according to the median value of neutrophil-to-lymphocyte ratio (cohort 1)

Factors	ALL (n=323)		*P*
Neutrophil-to-lymphocyte ratio	<2.43 (n=160)	≥2.43 (n=163)	
Age (years at diagnosis)			0.03
≥50	120 (75.00%)	103 (63.19%)	
<50	40 (25.00%)	60 (36.81%)	
Pathology			0.84
Adenocarcinoma	9 (5.62%)	11 (6.75%)	
Adenosquamous cell carcinoma	5 (3.12%)	7 (4.29%)	
Carcinoma	2 (1.25%)	1 (0.61%)	
Squamous cell carcinoma	144 (90.00%)	144 (88.34%)	
FIGO stage			0.002
- IB–IIB	53 (33.1%)	33 (20.3%)	
- IIIA–IIIC1	93 (58.1%)	97 (59.5%)	
- IIIC2–IVB	14 (8.8%)	33 (20.3%)	
Pre ALC (cells/μL)			0
≥1884	117 (73.1%)	43 (26.4%)	
<1884	43 (26.9%)	120 (73.6%)	
Min ALC (cells/μL)			0
≥276	97 (60.6%)	66 (40.5%)	
<276	63 (39.4%)	97 (59.5%)	
a2=a1+e1 (cells/μL)			0
≥1629	100 (62.5%)	62 (38.0%)	
<1629	60 (37.5%)	101 (62.0%)	
e1 (cells/μL)			0.133
≥144	88 (55.0%)	75 (46.0%)	
<144	72 (45.0%)	88 (54.0%)	
α			1
≥0.08	83 (51.9%)	84 (51.5%)	
<0.08	77 (48.1%)	79 (48.5%)	

FIGO, International Federation of Gynecology and Obstetrics; ALC, absolute lymphocyte count; Pre ALC, ALC before treatment; Min ALC, minimum ALC during radiochemotherapy; a2, estimated pre ALC; e1, estimated ALC nadir

Supplementary Methods

Plasma preparation & storage

In principle, blood was collected in an EDTA tube (purple stopper), stored at 4℃, and centrifuged within 2 h at 13,000 rpm and 4℃ for 10 min. The uppermost yellow layer of the centrifuged blood was divided at 300 μl into sterilized cryotubes and then the tube cap was labeled according to the assigned blood resource number. For long-term plasma storage, the conditions are pp tube, 0.5–2 ml, and (-85)~(-60)℃.

The following paragraphs describe the process of plasma exosomal RNA sequencing

Small RNA library construction & sequencing

Exosome was isolated from human plasma by mixing the plasma with Exo2D RNA solution (Exosomeplus). The detailed isolation process was performed according to the manufacturer's instructions attached separately. RNA from plasma-derived exosomes was extracted using the miRNeasy Serum/Plasma Kit (Qiagen, Valencia, CA) according to the manufacturer's instructions attached separately. The extracted RNA concentration was calculated by Quant-IT RiboGreen(Invitrogen). RNA size was confirmed using Agilent RNA 6000 Pico Kit and Small RNA Kit on Agilent 2100 Bioanalyzer (Agilent Technologies, Böblingen, Germany). The 10ng of RNA isolated from each sample was used to construct sequencing libraries with the SMARTer smRNA-Seq Kit for Illumina, following the manufacturer's protocol. Briefly, Input RNA is first polyadenylated in order to provide a priming sequence for an oligo(dT) primer. cDNA synthesis is primed by the 3' smRNA dT Primer, which incorporates an adapter sequence at the 5'end of each RNA template, it adds non templated nucleotides which are bound by the SMRT smRNA Oligo-enhanced with locked nucleic acid (LNA) technology for greater sensitivity. In the template-switching step, PrimeScript RT uses the SMART smRNA Oligo as a template for the addition of a second adapter sequence to the 3'end of each first-strand cDNA molecule. In the next step, full-length Illumina adapters (including index sequences for sample multiplexing) are

added during PCR amplification. The Forward PCR Primer binds to the sequence added by the SMART smRNA Oligo, while the Reverse PCR Primer binds to the sequence added by the 3’ smRNA dT Primer. The amplified libraries were purified from 6% Novex TBE-PAGE gels (Thermo Fisher, MA) to excise over than 138 bp (over than 18 bp of cDNA plus 120 bp of adaptors) fraction. Resulting library cDNA molecules include sequences required for clustering on an Illumina flow cell. The libraries were gel purified, and validated by checking the size, purity, and concentration on the Agilent Bioanalyzer. The libraries were quantified using qPCR according to the qPCR Quantification Protocol Guide (KAPA Library Quantificatoin kits for Illumina Sequecing platforms) and qualified using the TapeStation D1000 ScreenTape (Agilent Technologies, Waldbronn, Germany). The libraries were pooled in equimolar amounts, and sequenced on an Illumina HiSeq 2500 (Illumina,San Diego, USA) instrument to generate 51 base reads. Image decomposition and quality values calculation were performed using the modules of the Illumina pipeline.

Adapter trimming

The raw sequencing reads of small RNAs from the different experimental samples were pre-processed and analyzed with miRDeep2. Adapter trimming processed is done using cutadapt program to eliminate the adapter sequences that exist in the read which were attached to the miRNA during the smRNA library construction process. The first 3nt of all reads were trimmed to remove extra bases inserted during the SMART template-switching activity process. The adapter sequence and everything 3’ of the adapter were also removed. If a read matches more than at least first 5 bp of 3' adapter sequence, it was regarded the sequence truly is adapter sequence, and then trimmed from the read. Trimmed reads should be at the minimum of 18 bp in order to be considered reliable for analysis. Then, the remaining reads are classified into non-adapter reads, if they are not sequenced adapter sequences. In this analysis, trimmed and non-adapter reads were combined and regarded as processed reads for downstream analysis.

Clustering

To minimize the sequence uniqueness and computational intensity, adapter sequence processed reads are gathered and form a cluster. This cluster contains reads that are 100% match to the sequence identity and read length and are given its temporary cluster ID and the number of reads it holds.

Ribosomal RNA filtering

Most of the RNA composition is known as rRNA. In order to eliminate the effect of large amounts of rRNA, the read was aligned to the 45S pre-rRNA and mitochondrial rRNA of Homo sapiens and matched.

mRNA expression profiling

The reference gene annotation for *Homo sapiens* (GRCh38; release 109.20190607) was retrieved from NCBI. Since the produced read contains not only small RNA but also mRNA, mRNA expression profiling was performed using RSEM (v1.3.1) with options (--estimate-rspd --seed-length 15 --strandedness forward).

Identification of known miRNA reads

Sequence alignment and detection of known and novel microRNAs were performed using miRDeep2 software algorithm. Prior to performing sequence alignment, the *Homo sapiens* reference genome was indexed using Bowtie (1.1.2), a bowtie for aligning sequencing reads to reference sequences. Those reads were then aligned *Homo sapiens* matured and precursor miRNAs obtained from miRBase v21. The miRDeep2 algorithm is based on the miRNA biogenesis model; it aligns reads to potential hairpin structures in a manner consistent with Dicer processing, and assigns scores that represent the probability that hairpins are true miRNA precursors. In addition to detecting known and novel miRNAs, miRDeep2 estimates their abundance.

Proportion of miRNA and other RNA categories

Uniquely clustered reads are then sequentially aligned to reference genome, miRBase v21 and non-coding RNA database RNAcentral release 10.0 to identify known miRNAs and other type of RNA.
